# Adverse Effects of Cannabis Use and Associated Biomarkers: A Narrative Review

**DOI:** 10.1192/j.eurpsy.2026.10428

**Published:** 2026-07-31

**Authors:** J. Chapelas, L. Costa, M. Alves, J. Oliveira, A. Marois, N. Gimadeeva, R. Navarro

**Affiliations:** 1 Medical, Unidade Local de Saúde de Lisboa Ocidental (ULSLO); 2 Psychiatry, Unidade Local de Saúde São José; 3 Medical, Faculdade de Medicina Universidade de Lisboa, Lisbon, Portugal; 4 Medical, Faculty of Medicine, University of Montreal, Montreal, Canada; 5 Medical, I.M. Sechenov First Moscow State Medical University, Moscow, Russian Federation; 6 Psychiatry, Hospital da Luz Lisboa, Lisbon, Portugal

## Abstract

**Introduction:**

The expanding use of cannabis, in both recreational and medicinal contexts, highlights a dual reality of therapeutic potential and clinical concern. On one side, cannabinoids show promising medical applications, including chronic pain relief and antiemetic effects. On the other, cannabis consumption is linked to a broad spectrum of adverse outcomes. Psychiatric effects range from psychotic manifestations — such as cannabis-associated psychosis (CAPS) and earlier onset of primary psychosis in vulnerable individuals — to non-psychotic consequences including anxiety, amotivational syndrome and cognitive impairment. This heterogeneity of outcomes underscores the urgent need for reliable biomarkers to stratify individual vulnerability and to inform safer clinical practice.

**Objectives:**

To synthesise evidence on clinical, genetic, inflammatory, neuroimaging, and cognitive biomarkers linked to psychiatric (psychotic and non-psychotic) and non-psychiatric adverse effects of cannabis use.

**Methods:**

A narrative review of the literature was conducted. The databases PubMed and MEDLINE were searched using the terms *“Cannabis, Biomarkers, Psychosis, THC/CBD, Schizotypy, Psychotic vulnerability, Substance-induced psychoses”.* Inclusion criteria comprised studies reporting clinical, genetic, cognitive, or neurobiological data relevant to the risk of psychosis or other adverse outcomes related to cannabis exposure. Studies exclusively involving individuals with established psychotic disorders were excluded.

**Results:**

Findings converge on three main points. First, cannabis-related adverse effects are diverse and dose-dependent. CAPS rates are high in experimental THC challenge studies (~20%) but substantially lower in medicinal contexts with titrated THC+CBD (~1.5%). Non-psychotic psychiatric effects include anxiety, motivational deficits, and neurocognitive impairments. Second, individual vulnerability is influenced by genetic predisposition, inflammatory states, neurocognitive and oculomotor endophenotypes, and demographic factors. Third, promising biomarkers include polygenic risk scores, plasma protein panels, hippocampal responsivity, thalamocortical connectivity, and multivariate clinical-cognitive algorithms, though none are sufficient in isolation.

**Conclusions:**

Cannabis use can induce a spectrum of psychiatric and non-psychiatric adverse effects, with risk shaped by dose, formulation, and individual susceptibility. Risk stratification requires a multimodal approach that integrates clinical, genetic, inflammatory and neuroimaging measures. These insights should guide personalised counselling, safer medicinal protocols, and the development of stratified prevention and intervention strategies.

**Disclosure of Interest:**

None Declared

